# Construction of Discrete Model of Human Pluripotency in Predicting Lineage-Specific Outcomes and Targeted Knockdowns of Essential Genes

**DOI:** 10.1038/s41598-018-29480-w

**Published:** 2018-07-23

**Authors:** Priyanka Narad, Lakshay Anand, Romasha Gupta, Abhishek Sengupta

**Affiliations:** Amity Institute of Biotechnology, Amity University, Uttar Pradesh, India

## Abstract

A network consisting of 45 core genes was developed for the genes/proteins responsible for loss/gain of function in human pluripotent stem cells. The nodes were included on the basis of literature curation. The initial network topology was further refined by constructing an inferred Boolean model from time-series RNA-seq expression data. The final Boolean network was obtained by integration of the initial topology and the inferred topology into a refined model termed as the integrated model. Expression levels were observed to be bi-modular for most of the genes involved in the mechanism of human pluripotency. Thus, single and combinatorial perturbations/knockdowns were executed using an *in silico* approach. The model perturbations were validated with literature studies. A number of outcomes are predicted using the knockdowns of the core pluripotency circuit and we are able to establish the minimum requirement for maintenance of pluripotency in human. The network model is able to predict lineage-specific outcomes and targeted knockdowns of essential genes involved in human pluripotency which are challenging to perform due to ethical constraints surrounding human embryonic stem cells.

## Introduction

Human embryonic stem cells (hESC) are the cells having the potential to self-renew and remain viable for a long duration^1^. Due to these features, they are remarkably powerful source for studying early development and clinical treatments of a number of diseases^2^. In order to understand the complexities associated with the maintenance of pluripotent state in hESC and to harness the utility of stem cells as therapeutics, there is an urgent need to comprehend the overall cross-talk between transcriptional, epigenetic and signalling components involved in the process^3^. Human pluripotency is maintained by a complex interplay of a number of intrinsic and extrinsic factors^4^. The core transcriptional factors activate the pluripotency network by activating the genes responsible for maintenance of pluripotency and repressing the lineage associated genes^5^. Due to the preceding advantages, a plethora of high throughput studies have been conducted on hESC such as cDNA microarrays^6^, RNA-seq^7^, ChIP-seq^8^, immunoprecipitation followed by mass spectrometry (IP-MS) proteomics^9^ and inhibitory RNA (RNAi) screens^10^ to name a few. Nevertheless, integrating the huge amount of datasets into a systems level regulation is still a challenge that needs to be addressed. Static network diagrams are useful in providing a comprehensive map of the big-picture; still, it is imperative to develop discrete models of regulation that would be able to record accurately the behaviour of cell fate decisions over time^11^.

Single cell heterogeneity is common in pluripotent stem cells and previous studies have exhibited statistical and bioinformatics methods^12–15^. For example, Dowell *et al*.^16^ incorporated the gene expression, protein-protein interactions, ChIP-seq, RNAi screens and epigenetics markers to construct a Bayesian model of pluripotency-associated genes. The main aim of their work was a comparison of human and mouse embryonic cells. Further, their networks models are static. However, the major advantage of this approach can be that network does not need to be determined by a priori which allows the invention of novel self-renewal and pluripotency components. Lee and Zhou^17^ combined ChIP-seq, gene expression and motif finding data for the identification of transcription factors that can synergistically work together within the pluripotency circuitry. With this, they were able to establish 27 interactions among 14 factors. We observed that a number of these interactions are consistent with our study. In one of the other similar study Dunn *et al*.^18^ constructed data constrained Boolean model which connected 12 transcription factors among 16 interactions that suggest the least circuitry required to maintain pluripotency of mESC. Notably, Xu *et al*.^19^ also, constructed a directional network of 30 pluripotency genes that are useful in predicting stem cell fate decisions. In comparison to these studies, we have considered data from total 1018 single cells from snapshot progenitors and 758 single cells from time course profiling.

In this work, we utilize a discrete modelling approach to identify novel regulators of the human pluripotent network by utilizing gene knockout experiments together with Boolean based modelling. Boolean modelling is optimally the most appropriate for a large number of nodes where edges would represent regulation of the nodes taken from perturbation datasets^20^. Here we present a three-step methodology where (i) first, we construct a directed network from the previous work on human pluripotency network^21^. This network data consisted of a manually curated network which consists of all the elements involved in the maintenance of human pluripotency from 147 publications and the network consists of 122 human genes/proteins. We filtered the previous network for those specific links which depicted activation/inhibition only. Manual curation gives us direct evidence for the directionality of the nodes/edges, (ii) second, we studied the logic of the network using the single cell gene expression data, (iii) third, we performed combinatorial knockdowns of specific nodes which were instrumental in the maintenance of human pluripotency to predict the role of important regulators (positive/negative) of pluripotency. Our computational perturbation experiments revealed a new set of interesting putative pluripotency regulating genes.

## Results

### Construction of a signed network of human pluripotency

**Figure 1:**
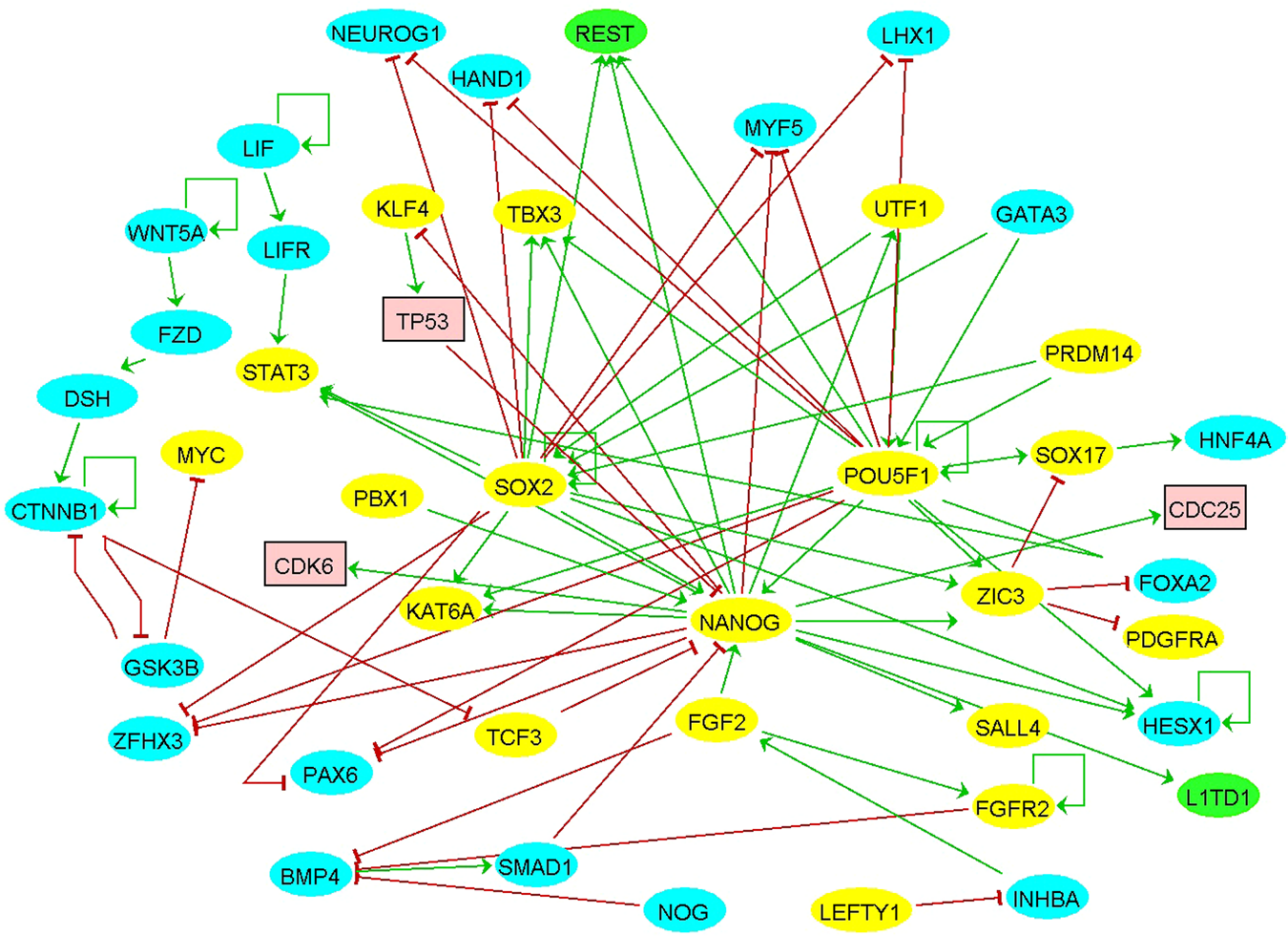
A 45-node signed directed network constructed manually from literature. Inhibitions and activations are represented as red T-bar and green arrow respectively. Nodes/genes are classified as pluripotency transcription factors (oval yellow), differentiation genes (oval blue), epigenetic factors (oval green), and others (pink squares).

The condensed network of human pluripotency was extracted from previously constructed human pluripotency network. This condensed network consists of 19 transcription factors, 21 differentiation genes, and 2 epigenetic factors. This network was developed by manually adding the nodes (genes/proteins) and the edges (activations, interactions and inhibitions) that are reported in the literature showing direct mechanisms involved in the induction and maintenance of pluripotency in the human model system. The criteria for inclusion of nodes and the edges were restricted: nodes and links added must be directly involved in induction and maintenance of pluripotency and in the human model system. This inclusion criterion ensures the quality of the network and also prevents it from unnecessary expansion. The network layout was produced by manually adding nodes and edges. The interactions in the human pluripotency network were extracted from the criterion of loss of function or knockdown of the transcription factors and epigenetic factors. The 45 nodes included have pluripotency regulators for which literature evidence was available and the same approach was utilized for identification of differentiation genes. The list of studies and the criterion of inclusion in the network is provided in Supplementary File 1. The layout includes nodes and edges where the nodes represent genes or gene products (i.e. proteins). Two types of mechanisms were considered for the edges: (i) Firstly, activation is denoted by an arrow and (ii) Secondly inhibition is denoted by a T-bar. The final network consists of 45 nodes, 65 edges and 4 positive auto-regulatory loops [Fig. 1].

### Measurement of expression levels in hESC

**Figure 2:**
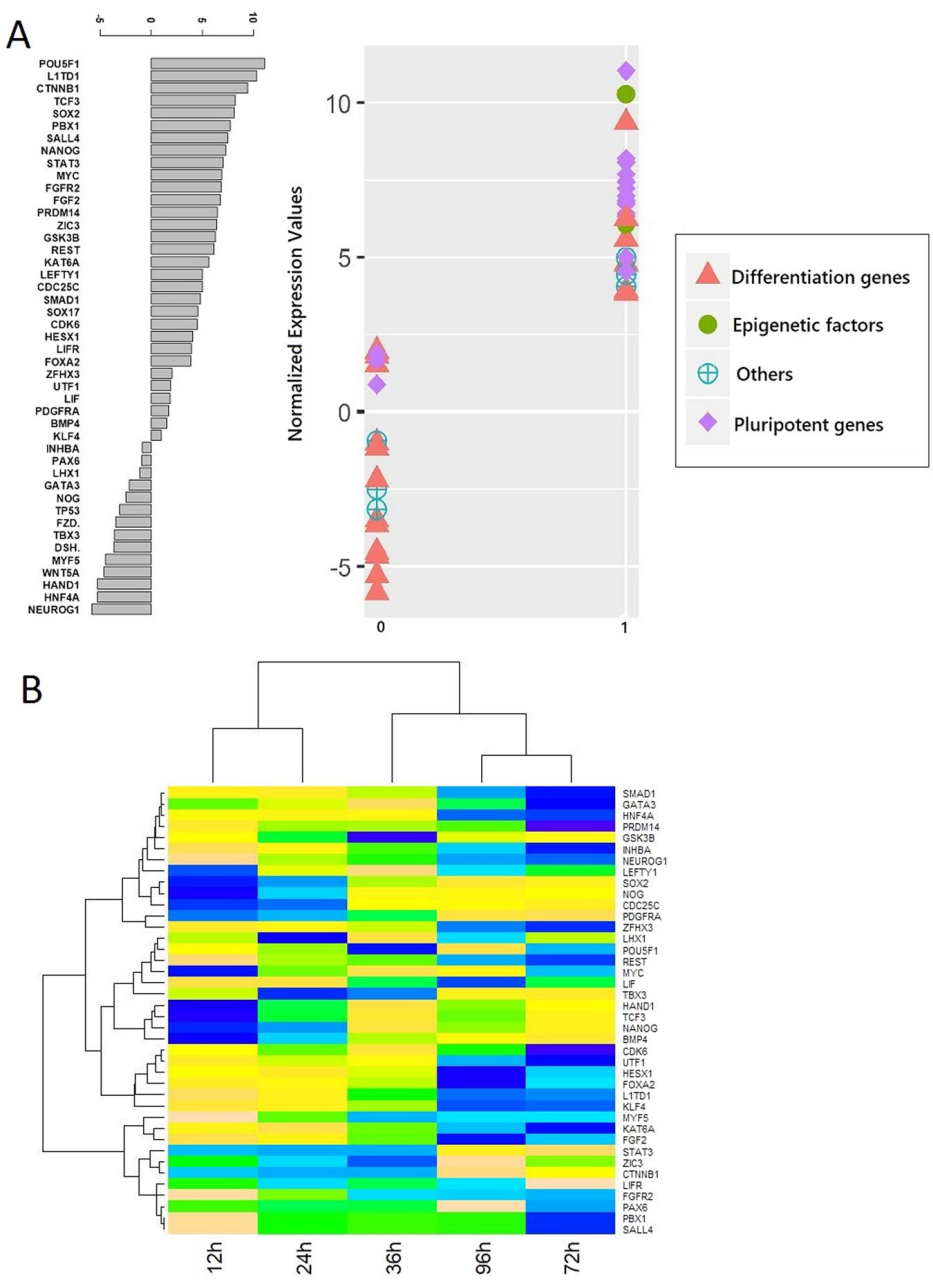
(**A**) Binarization of the expression values of the 45 genes using k-means clustering. Majority of differentiation genes are present in the high-expression cluster (assigned value 1) while most of the pluripotent genes are present in the low-expression cluster (assigned 0). (**B**) Histogram for the time-series RNA-seq expression of hESC. Columns signify the time intervals while rows represent the 45 genes in the network.

The initial network topology consisting of 45 nodes was used to identify novel links as described in the sections below; however, the network abstraction cannot explain the essence of regulation in the real world. The coding of regulation of transcription into a Boolean logic gate can be considered as mathematical idealization and the abstraction of complex metabolic and biochemical processes of regulations of transcription. In practice, Boolean modeling is able to depict the importance of the regulatory mechanisms but still needs validation through a time series data. Expression data was retrieved from Gene Expression Omnibus^22^. The hESC transcriptome data that we have used is single-cell RNA-Seq expression profiling of 1018 single cells from snapshot progenitors and 758 single cells from time course profiling performed using Illumina HiSeq 2500^23^. The data is present in the form of count matrix consisting of reading counts for 19,097 genes that, further, entails pruning to include only 45 genes present in our network. The preliminary task, therefore, was to normalize the data which was performed using R limma package (logCPM normalization)^24^. The normalized data is provided as Supplementary File 2. The concept of Boolean networks is based on the fact that the expression of genes in a gene regulatory network exhibits bimodality, that is, genes are expressed only above a threshold expression value. We have bifurcated the expression values of the 45 genes into two clusters by applying k-means clustering algorithm. This facilitates binarization of data as we assigned a ‘1’ to the high expression value cluster while a ‘0’ to the lower one [Fig. 2A,B]. Major Pluripotent genes such NANOG, POU5F1, SOX2, MYC, FGF2 are present in the cluster with high expression values (assigned as 1). While most of the differentiation genes such as LIF, WNT5A, HAND1, HNF4A, NEUROG1, GATA3 are present in low expression cluster (assigned as 0). Epigenetic factors are also present in high expression cluster as is evident from the clustering results. Most pluripotent genes land on one side of the K-means threshold while most of the differentiation genes land on the other side. It means there is dependence between the binary values and the category of genes. This was further confirmed by performing chi-square test of independence. The observations were as follows:

1 $${\rm{Pearson}}\mbox{'}{\rm{s}}\,\mathrm{Chi}-\mathrm{squared}\,{\rm{test}}:\,p-\mathrm{value}=0.02131$$

**Figure 3:**
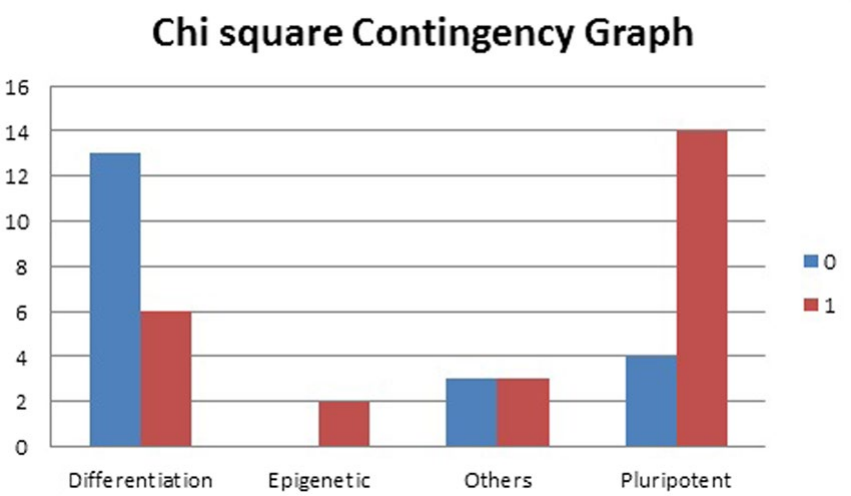
Chi-square test graph. Majority of differentiation genes are present in the high-expression cluster (assigned value 1) while most of the pluripotent genes are present in the low-expression cluster (assigned 0).

As p-value is 0.02131 which is less than the significance level of 0.05, we reject the null hypothesis and conclude that the two variables are in fact dependent. Thus, we can infer from the p-value and the contingency graph [Fig. 3] that, it is apparent most of differentiation genes are in binary state ‘0’ while most of the pluripotent genes are in binary state ‘1’. However, for the unimodal genes (whose time course expression did not show two humps) cannot be assigned binary state based on their modality. For those unimodal genes, we performed k-means clustering on their single expression value (non-time series data) with k = 2, hence obtaining two groups for the genes. Genes in the higher group were assigned 1 while those in the lower group were assigned 0. Now each of the genes was assigned a binary state of 0 or 1. Then, we plot them and observed the pattern (that we confirmed with chi-square test).

Further, to validate the bimodularity of the genes, we utilized the modes package in R^25^. Modes package calculates bimodality amplitude. This is a measure of the proportion of bimodality and the existence of bimodality. The value lies between zero and one (that is: [0, 1]) where the value of zero implies that the data is unimodal and the value of one implies the data is two point masses. All non-zero values are considered as bimodal [Supplementary File 3]. The binary states were also used for the validation of the Boolean functions. Validation of the Boolean function means for e.g. when we put the observed values of the genes such as Gene A, Gene C and Gene D and calculate the logic value for B, we can compare the logic value of B with the observed value of B. For example, the binary state assigned for A, C, D is 0, 0, and 1 respectively, then the logic value of B, i.e. 0 & 0 | 0 is 0. Now if the assigned value of B is also 0 the function is validated. Around 93% of our Boolean function showed validation, which, we further used for analysis.

### Regulatory Logic of hESC network

The transformation of statichuman pluripotency network into a Boolean network was done by learning regulatory logic functions for each node. The detailed logic functions are provided in Supplementary File 4. Initially, a network consisting of 45 genes and 69 interactions was extracted by pruning of the original network. The interactions (activations or inhibitions) in the pruned network are obtained from the original topology of the network. The logic functions for each node, confined to only OR, AND and NOT logic gates, were manually added based on the interactions of nodes in the pruned network. Each logic function complies with the following rules: (1) All activators or inhibitors are connected by OR except for any interaction that has prior knowledge from literature (the dimer POU5F1-SOX2 was AND-connected). (2) Inhibitors and activators are connected to each other by AND logic. The set of rules are defined in Supplementary File. This ensures that the gene will be inhibited by any one active inhibitor despite several active activators^26^. The binarized expression data, produced through clustering, was used to corroborate the logic functions.

**Figure 4:**
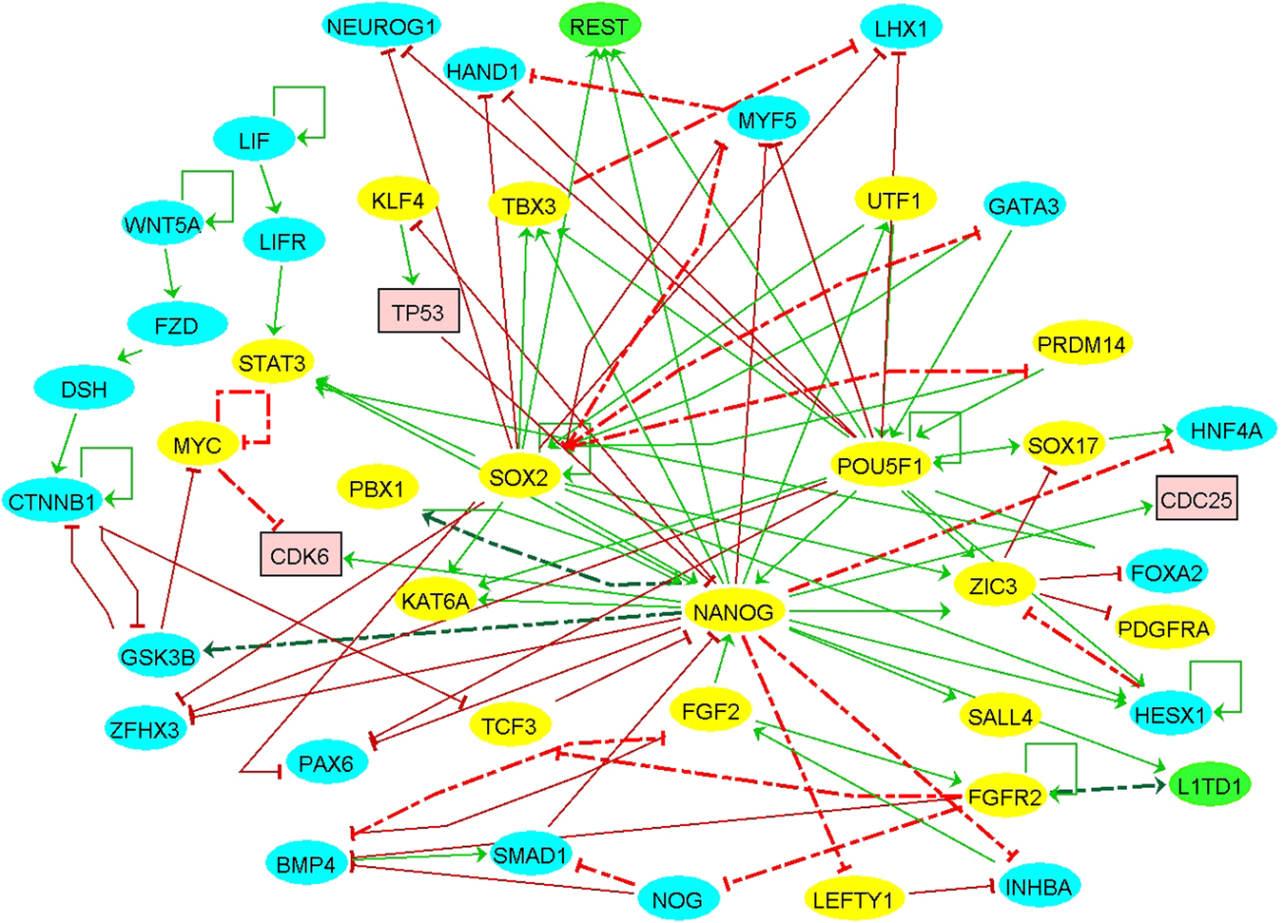
A 45-node signed directed integrated network including novel interactions learned from the reconstructed network (obtained from time-series RNA-seq data). Novel interactions are represented by dashed red T-bar (inhibition) and dashed green arrow (activation).

Interestingly, each logic function satisfied the input and output relationships. Prior to further analysis of the network, certain refinements were made in the network. The normalized time-series expression data for the hESC was used to reconstruct a Boolean network using the BoolNet software (an R package)^27^. The software generates several plausible logic functions for each node that would produce myriad plausible Boolean networks. From the pool of the various plausible logic functions, those that are maximally similar to the one obtained by topology-based learning were manually selected for each node that leads to the generation of a single reconstructed Boolean network. Evaluation of the reconstructed network unearthed various novel interactions that were not present in the original network topology. Moreover, some undefined interactions from the original network were defined using the predicted interactions of the reconstructed network. The original network was refined by adding these novel interactions. The resultant network referred to as integrated network [Fig. 4], was used for further simulation and perturbation experiments. Based on the refined topology of the integrated network, logic functions were manually constructed for each node of the network.

The integrated network encompasses the consensus and unique interactions. Interactions for KLF4 and TBX3^28^, which was not specified in the original network, were predicted from the reconstructed network and added to the integrated network. As predicted from the reconstructed network, interestingly, KLF4 is inhibited by NANOG while TBX3 is activated by NANOG, POU5F1, and SOX2^29^. Some novel interactions pertaining to important cell-signalling factors MYC, BMP4, and FGF2 were also included in the integrated network. FGFR2, which is activated by FGF2, inhibits TCF3, NOG, and BMP4^30^. The signalling factor, c-MYC, exhibits a negative auto-regulatory loop^31^. Moreover, negative and positive feedback loops are identified in the integrated network which is essential for homeostasis.

### Single and combinatorial perturbations of important pluripotency maintenance genes

The integrated network consisting of 45 network nodes with logic applied can now be subjected to simulations and perturbations. These perturbations can further be subjected to experimental validations. Towards this end, we first performed the single perturbations of the core pluripotency circuit genes (POU5F1, NANOG, SOX2) and some more genes touted to be important for induction/loss of human pluripotency (L1TD1, KLF4, UTF1, FGF2, BMP4, and GSK3B) and further performed important double and triple knockdowns. The trained Boolean model was utilized for making perturbations and studying the effect of perturbations. Computationally simulations were done by forcing a gene node to be in a stable OFF (0) state. Beginning with 100 random initial conditions, step-wise perturbations were performed and stable state was achieved for each node in the network.

**Figure 5:**
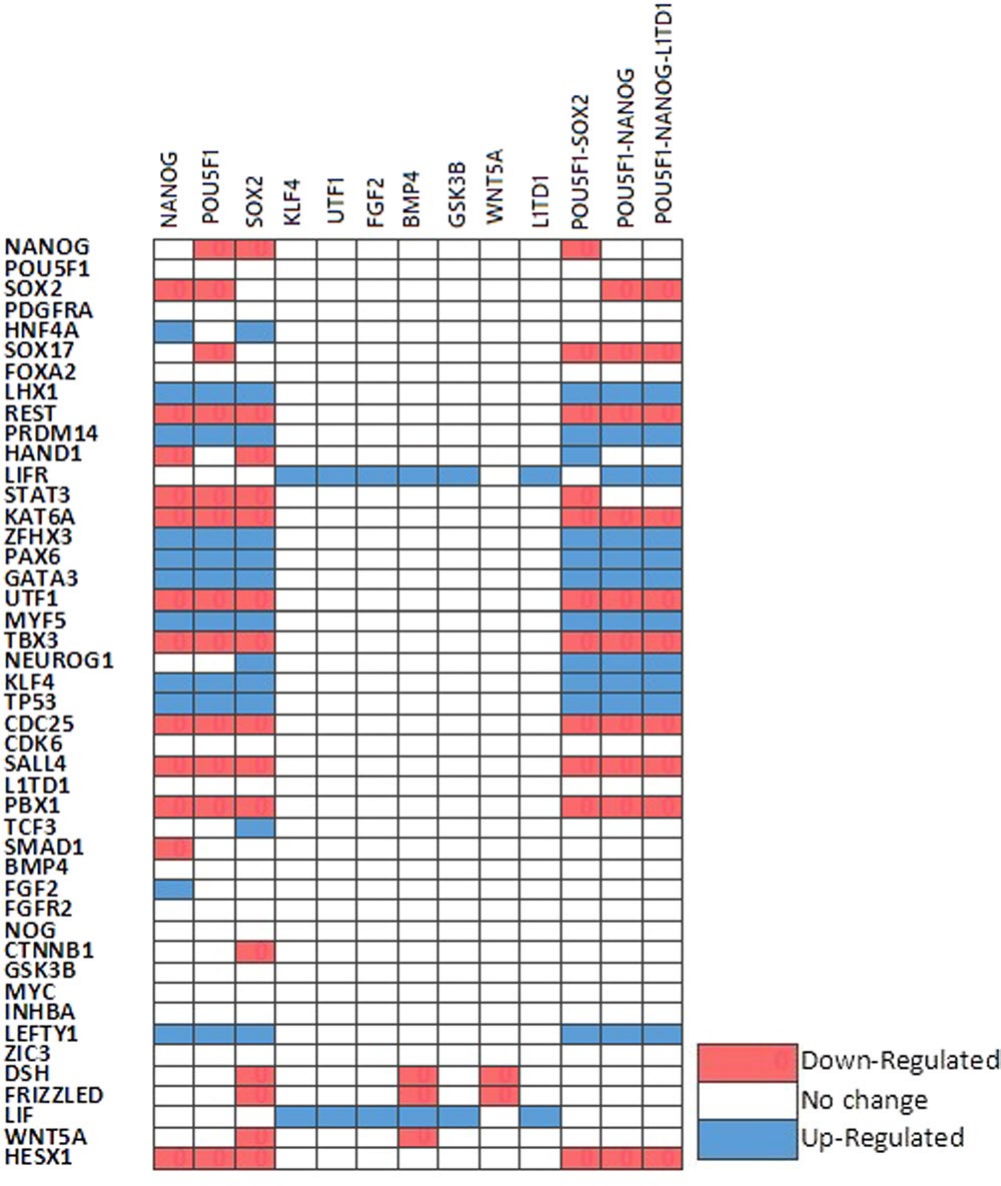
Individual and combinatorial *in silico* perturbation results for the selected genes. Downregulation and up-regulation are depicted as red box and blue box respectively. The genes that were unaffected by the perturbations are represented as a white box.

Each of the single/combinatorial *in silico* perturbations repressed the core circuit of hESC pluripotency and activated selective differentiation markers which are in congruence with the previous experimental studies^5^. We also performed perturbations that led to a steady state or we define them as the minimum requirement for maintenance of pluripotency in human. It was observed that not all the core pluripotency genes were repressed but selective repression took place. We have performed 10 single and 3 combinatorial knockdowns of genes involved in pluripotency. We have explained only the knockdowns of POU5F1 & NANOG in the main text, which are instrumental in maintenance of pluripotency. Also, we discuss the knockdown effects of combinatorial knockdowns which are important to understand the minimum requirement of maintenance of pluripotency. Rest of the *in silico* perturbations are depicted in Fig. 5.

Knockdown of NANOG leads to shut down of core ES transcription factors such as SOX2, STAT3, and HESX1. While the differentiation genes were upregulated, others including LIF and LIFR were downregulated. Knockdown of NANOG also leads to the downregulation of epigenetic factor REST. With experimental reports previously it has been proved that REST is highly abundant in ES cells and functions in part to repress neuronal-specific genes^32^. Interestingly, the shut-down of NANOG has no effect on POU5F1 indicating that NANOG may not be a direct regulator of POU5F1 regulation. Knockdown of NANOG leads to up-regulation of lineage-specific genes belonging to the endodermal specification. Individual perturbation of POU5F1 resulted in down-regulation and up-regulation of 16 genes. Knockdown of POU5F1 leads to shut down of the core circuit of ES cell pluripotency genes advocating its role as the master regulator of human pluripotent stem cells^33^. Also, down-regulated was INHBA, which is member of the TGF-beta (transforming growth factor-beta) superfamily of proteins, which reports the importance of TGF-beta signalling pathway in the maintenance of pluripotency in human. Further, POU5F1 perturbation led to activation of lineage-specific genes belonging to the mesodermal specifications.

Knockdown result for the dimer POU5F1-SOX2 is similar to that for POU5F1 alone except for it didn’t result in downregulation of LIF and LIFR. Knockdown results for the combination POU5F1-NANOG are exactly similar to that of POU5F1 alone. The RNA-binding protein L1TD1 is one of the most specific and an abundant protein in pluripotent stem cells and is essential for the maintenance of pluripotency in human cells^34^. In order to identify and establish its role in human pluripotency, we performed its perturbations in for the combination POU5F1-NANOG-L1TD1 and observed that L1TD1 could activate only POU5F1 and KLF4 but was not able to directly turn on NANOG and SOX2.

The next step was to check the prediction accuracy of the model with the help of single-cell data and our initial network topology. Each of the *in silico* knockdown performed, the computational knockdown result is supported/compared with previous experimental knockdowns. In general, the Boolean model predictions are touted to be reliable; we performed perturbations for possible components involved in the maintenance of human pluripotency. On the whole, the Boolean model created can predict the effects of transcription factor perturbations on lineage commitment. This model can be highly useful to predict more interesting knockdowns which cannot be easily tested through high throughput experiments because of ethical constraints surrounding the human embryos.

## Discussion

In this work, a combination of logic rules was used and reconstructed a Boolean Network to create an integrated network of human pluripotent stem cells. Within the last decade, after the identification of core pluripotency circuit^5^, a number of pluripotency networks have been proposed^35,36^. All these networks gave us a comprehensive framework of important interactions for induction/maintenance of human pluripotency but were static and descriptive in nature. In this study, we were able to construct a discrete model of hub genes of human pluripotency that can be subjected to perturbations to predict the effect of gene knockouts of regulatory genes and their corresponding targets. By constructing this model, we facilitate the scientist to perform *in silico* single/combinatorial knockouts, which would be challenging to perform for the human system *in vitro* or *in vivo*. Perturbation experiments *in silico* have been performed previously on expanding human pluripotency in the past^37^, however, it is to our knowledge the first logic-based model of hub genes involved in human pluripotency and thus would be an ideal predictive model for studying complexities associated with human pluripotency.

Perturbations are very useful for the identification of the effect of knockdown of a gene. However, all single and combinatorial knockdowns are not possible as some of the proteins can be difficult to alter or miRNA are not known, hence *in silico* models can provide a nearly optimal solution for an understanding complex phenomenon like pluripotency. In this work, the network model was validated by performing single, double and triple knockdowns which are quite challenging *in vitro*. We observed that the critical factors governing pluripotency (OCT4, SOX2, and NANOG) are expressed in a steady state of hESC^38,39^. Other important factors such as major signalling pathways (BMP4, FGF2) and epigenetic factors (REST) are also expressed^40^. As expected, BMP4 knockdown led to the stable expression of the steady state and hence maintenance of human pluripotency^41^. Also, it was observed that knockdown of BMP4, led to up-regulation of FGFR2. FGFR2 is an important regulator of hESC pluripotent state and its knockdown induces differentiation^42^. Interestingly, knockdown of NANOG and POU5F1 led to the downregulation of SOX2^43^. It was inferred from this observation that SOX2 may not be a crucial factor for independent regulation of human pluripotency and may be dependent on its interaction with OCT4/NANOG^44^. Individual knockdown of NANOG led to up-regulation of lineage-specific genes pertaining to endodermal specifications. Individual knockdown of POU5F1 had major implications on the core pluripotency circuit downregulating both NANOG and SOX2, shutting down the core circuit of pluripotency completely^45^. Down-regulation of POU5F1 leads to the upregulation of mesodermal genes, which are lineage-specific genes. UTF1 expression shuts down with POU5F1 leading to a suggestion that UTF1 is strongly regulated by core circuit genes^46^. Similar results were observed for SOX2 knockdown. Another significant knockdown was that of L1TD1. It was expected a major shut down pertaining to differentiation genes. Surprisingly, it was observed that single perturbation of L1TD1 was unable to produce any significant knockdown of pluripotent/differentiation genes. Combinatorial knockdown was then performed with POU5F1-NANOG-L1TD1. This combination knockdown resulted in maximum disruption of pluripotency genes and activation of differentiation genes. It was inferred from this observation that L1TD1 is an integral part of the interactome network of core circuit of POU5F1, SOX2 and NANOG^34,47,48^. Our knockdowns resulted in significant congruence with literature. Altogether this study shows that the combined approach of systems biology and experimental biology can predict and identify factors that are counterintuitive and, hence, hard to discover *in vitro*. Moreover, this approach can be significantly applied to other cellular system and thereby enhance research progress and newer insights.

## Methodology

### Identification of hub genes

In our previous work, we constructed an extensive network of human pluripotency^21^. This gene regulatory network was then filtered for the genes belonging to three categories only, viz. pluripotency genes, differentiation genes, and epigentic factors. The resultant network was considered as base network with original network topology. The original network consisted of 122 genes and 166 interactions. The network was pruned for the hub genes involved in induction/maintenance of pluripotency for which RNA-seq data^7^ was available in case of hESC. The initial literature network was expanded using gene perturbation data from Gene Expression Omnibus. The complete list of references is provided in Supplementary File 1.

### Regulatory Logic

Based on the original network topology, Boolean functions were manually constructed for each gene in the network (except for the input genes). The Boolean functions were defined using the formula along with modifications based on whether the genes are co-expressed or not. Boolean logic was first described long back as a source of biological modeling in the 1960s^49^. In our network, for each node I in the network, a Boolean function Ψ_i_ comprising of only AND, OR, and NOT logic gates were manually constructed. Each Boolean function complies with the following rule:

2 $${\psi }_{{\rm{i}}}=({{\rm{A}}}_{1}|{{\rm{A}}}_{2}|{{\rm{A}}}_{3}\ldots |{{\rm{A}}}_{{\rm{n}}})\,\& \,({{\rm{I}}}_{1}|{{\rm{I}}}_{2}|{{\rm{I}}}_{3}\mathrm{..}|{{\rm{I}}}_{{\rm{n}}})$$

where all activators A and all inhibitors I for the node i are connected through | (OR) logic gate while they are connected to each other by & (AND) logic gate. Inhibitors are represented by! (NOT) in the Boolean function. One exception to the rule is the dimer POU5F1-SOX2 which is always AND-connected. The normalized time-series RNA-seq expression data was used for the *in silico* reconstruction of the network comprising 45 nodes using BoolNet, R package. A reverse engineering approach was used to construct an *in silico* GRN consisting of the same genes as the original network. For this, time-course RNAseq expression data was used. Using heuristic search algorithms, BoolNet uses a time-course expression data, to compute various plausible Boolean functions for each gene in the network. Each predicted function has the same probability value.

From the pool of predicted Boolean functions for each node, the Boolean functions were manually picked which possesses maximum similarity with the topology-based learned Boolean functions i.e. if a predicted Boolean function consists of the genes that are related in the original network. Using the manually picked Boolean functions, we constructed an *in silico* GRN consisting of the same genes. Interestingly, most of the interaction in the predicted network was same as that of the original network. However, our reconstructed network was able to predict novel interactions in the network learned from the predicted network. Also, some interactions were not defined in the original network, i.e. it was not known whether the interaction is stimulation/inhibition. Those interactions were predicted from the reconstructed network. Hence, the final network, which we refer to as the integrated network, is the original network modified after inclusion of novel learned interactions.

### Bimodality Testing

The normalized single-cell RNA-seq expression data was binarized for the validation of the Boolean functions. This was performed by 3 different statistical approaches. Binarization was first performed by applying K-means clustering algorithm with K = 2 (Binarize, R package) engendering bifurcation of the data into two clusters. The clusters produced are such that similar data are included in the same clusters. We performed k-means clustering on expression data so that genes are grouped on the basis of expression values. K = 2 is used to form two groups so that to assign two binary states to the cluster. The clusters with high expression values were assigned a 1, while that with low expression value is assigned 0. K-means is more robust to groups with very different sizes than arbitrary threshold such as the median or a quartile and it’s more straightforward than having to guess an arbitrary value.

A threshold value ρ separating the two clusters was used to assign a binary state to the expression values of 45 genes. Expression values ε were assigned 1 for ε > ρ and 0 for ε < ρ. For the validation of Boolean function Ψ(t), logic values for each component in the Boolean function at t − 1 were inserted to obtain the logical output at t which is then checked with the expected output. Further, we use the chi-square test to cross check our observations from K-means clustering. For chi-square test, we used a null hypothesis stating that the random variables “binary values” and “gene category” are independent, and an alternate hypothesis stating that the two variables are dependent. The lines of code are appended in the supplementary file. Thirdly, we performed bimodality testing using modes R package.

### *In Silico* Simulations and Perturbations

The Boolean network that was constructed by logic-learning method was then subjected to *in silico* simulations and perturbations. Initially, state-transitions (S_t_ state of the network determined by S_t−1_) were tested for various combinations of initial states of the genes in the network that culminates into a stable state of the network. Minimum essentiality of genes that produces a culminating stable state of the network encompassing activated essential pluripotent transcription factors NANOG, POU5F1, and SOX2, were determined by activating selected gene(s) (setting its value to 1) in the initial condition of the simulation. For the *in silico* perturbation, the gene(s) were coerced to OFF state during the simulation of the Boolean network. The final stable state, called the attractor, was recorded for each gene perturbation. 100 random initial states were chosen for the simulation of the network in the synchronous mode. Many attractors with varying probabilities were generated. Each attractor A_i_ was assigned a weight B_i_ that represents its number of basins (number of initial states that leads to the attractor state). The attractor with maximum weight B_max_ was chosen as the final stable state. For each gene G_i_ in the network, the perturbation change S_i_ is determined as follows:

3 $${S}_{i}=\{\begin{array}{ll}\mathrm{up} \mbox{-} \mathrm{regulated} & {\rm{if}}\,{{\rm{G}}}_{{\rm{iu}}}={\rm{0}},\,{{\rm{G}}}_{{\rm{ip}}}={\rm{1}}\\ \mathrm{down}-\mathrm{regulated} & {\rm{if}}\,{{\rm{G}}}_{{\rm{iu}}}={\rm{1}},\,{{\rm{G}}}_{{\rm{ip}}}={\rm{0}}\\ {\rm{unchanged}} & {\rm{if}}\,{{\rm{G}}}_{{\rm{iu}}}={{\rm{G}}}_{{\rm{ip}}}\end{array}$$

where Giu and Gip are the states of the gene Gi in the unperturbed and perturbed networks respectively. Several individual and combinatorial *in silico* perturbations of nodes were performed to analyze the effect of knockdowns on the network. Such *in silico* predictions might be advantageous to predict the role and contribution of a gene in the determination of cell fate. Knockdowns of transcription factors NANOG, POU5F1, SOX2, epigenetic factors L1TD1, LHX1, and signalling factors BMP4, FGF2, WNT5A, and KLF4 was performed individually and in several combinations. The Boolean network is assigned initial state i.e genes are given a value of 0(inactivated) or 1(activated) for time t. The network is simulated subsequent time like t + 1, t + 2 … n until it reaches a stable state that is called as attractor. The attractor is believed to be the biologically stable state of the network. For our analysis, we simulated the network using BoolNet. It takes a file consisting of the list of Boolean functions. We tested the network for various initial states. We activated only essential genes like NANOG initially to see whether it triggers the entire network. For statistical accuracy, we considered 100 random initial states to obtain the attractor. Using the same package and network, we then performed single and combinatorial knockdowns to see its impact on the state of network. A gene is knocked (setting its state to 0) out that means during simulation of the network its state will never change. Now after, knocking down the gene, the same simulation step was performed to obtain the stable state (attractor) of the perturbed network. State of the normal network and that of the perturbed network were then compared to see the differences: [1] a change from 0 to 1 indicate up-regulation of that gene; [2] a change of 1 to 0 indicate down-regulation of that gene. A diagrammatic summary of the steps is provided in the supplementary file to enhance the readability and reproducibility of the work.

Source code to run the insilico simulations is provided in an open-source repository (GitHub) https://github.com/pnarad/hPluriNet-Boolean-Modelling. It contains supplementary R code file and one redme.md file which can be used for the reproducibility of the work.

## Conclusion

In this work, a predictive model of gene regulatory network of human pluripotency was developed consisting of 45 nodes on the basis of literature curation. Logic-based modelling was used to learn regulatory logic with the network nodes using the single cell RNA-seq data. The expression values in single cell showed some bimodality which was fitting well with the logic rules of the network. The significance and utility of logic modelling is its ability to be able to manipulate the network by performing *in silico* perturbations. The strong congruence between the discrete model and experimental knowledge gives us direct validation of the developed model and capture some significant essence of pluripotency in human. However, we would still be interested in further challenges associated with our network model. For example, our model is binary having used a common notation for gene/gene products assuming a direct role of transcription factor and their expression which may not be the case always. Further, pluripotency in human is also affected by other factors such as histone modifications, chromatin modifications, small molecules and miRNA which have not been explicitly included in the network due to the complexity and multi-dimensionality associated with these network components.

### Data availability

All the supporting data is provided as supplementary files.

## Electronic supplementary material

Dataset 1

Dataset 2

Dataset 3

Dataset 4

Supplementary File
