# Supplementary material for: Construction of Discrete Model of Human Pluripotency in Predicting Lineage-Specific Outcomes and Targeted Knockdowns of Essential Genes
Source: Sci Rep. 2018 Jul 23;8:11031. doi: 10.1038/s41598-018-29480-w (PMC6056480; doi:10.1038/s41598-018-29480-w)
Supplement: Supplementary file 5 — Supplementary File [file 41598_2018_29480_MOESM5_ESM.docx]

**Construction of Discrete Model of Human Pluripotency in Predicting Lineage-Specific Outcomes and Targeted Knockdowns of Essential Genes**

*Priyanka Narad^1*#^, Lakshay Anand^1#,^ Romasha Gupta^1^, Abhishek Sengupta^1#^*

^1^Amity Institute of Biotechnology, Amity University, Uttar Pradesh, INDIA

Email: [pnarad@amity.edu](mailto:pnarad@amity.edu)

**^*^Corresponding Author**

Priyanka Narad, Amity Institute of Biotechnology, Amity University, Uttar Pradesh, INDIA.

Email: [pnarad@amity.edu](mailto:pnarad@amity.edu)

Methodology

In silico Simulation and perturbations of integrated network using BoolNet.

Integrated Topology: adding some novel interactions from inferred topology to initial topology

Inferred Topology: Reconstructing a boolean network from time-series RNAseq data using BoolNet

Binarization of the normal data to assign values 0/1 that can be used to validate the boolean functions

Initial Topology: Constructing the boolean functions for each node based on the rules

Normalization of data using limma package to make it ready for analysis

Selecting the genes for initial network topology based on previous work

Collection of suitable time-series & normal RNAseq data filtered for only genes considered in the network
